# Child drowning in Indonesia: insights from parental and community perspectives and practices

**DOI:** 10.1093/heapro/daae113

**Published:** 2024-09-03

**Authors:** Muthia Cenderadewi, Richard C Franklin, Prima B Fathana, Susan G Devine

**Affiliations:** Department of Public Health and Tropical Medicine, College of Public Health, Medical and Veterinary Sciences, James Cook University, Bebegu Yumba Campus, 1 James Cook Dr, Townsville, Queensland 4811, Australia; Faculty of Medicine, University of Mataram, Jalan Pemuda Nomor 37, Mataram, West Nusa Tenggara 83127, Indonesia; Department of Public Health and Tropical Medicine, College of Public Health, Medical and Veterinary Sciences, James Cook University, Bebegu Yumba Campus, 1 James Cook Dr, Townsville, Queensland 4811, Australia; Research, Policy and Advocacy, Royal Life Saving Society—Australia, Suite 6, Level 4, 173-179 Broadway (Cnr Mountain St), Broadway, Sydney, New South Wales 2007, Australia; Faculty of Medicine, University of Mataram, Jalan Pemuda Nomor 37, Mataram, West Nusa Tenggara 83127, Indonesia; Department of Public Health and Tropical Medicine, College of Public Health, Medical and Veterinary Sciences, James Cook University, Bebegu Yumba Campus, 1 James Cook Dr, Townsville, Queensland 4811, Australia

**Keywords:** drowning, prevention, health promotion, community health promotion, health behaviour, community safety promotion, community engagement, low- and middle-income countries

## Abstract

Child drowning is a significant public health issue in Indonesia, however, there is insufficient understanding of the issue and its associated risk factors within communities. This qualitative study aimed to explore parental and community perceptions and practices related to child drowning in Indonesian communities, and the perceived causes and risk factors. Seven focus group discussions (*n* = 62) were conducted with parents of children aged under-5 years and village community leaders in seven villages across all districts of Lombok Island, West Nusa Tenggara Province of Indonesia. Participants were recruited using purposive and snowball sampling. The thematic analysis, guided by Braun and Clarke’s framework, used both deductive approaches, utilizing the Health Belief Model’s constructs and inductive approaches. Most participants were unaware of the susceptibility of their children and others in their community to drowning and of the potential severe outcomes of drowning such as injury, disability and death. Participants generally associated drowning with beaches or open seas. Unprotected wells, tubs and buckets were identified as notable risk factors for child drowning in and around the home, shaped by some experience of child drowning incidents in the community. Supervision was identified as protective factor, however, mothers were often unavailable to supervise children, and supervision responsibility was often delegated to other family and community members. This study highlights the urgent need to enhance public awareness regarding children’s susceptibility to drowning. Further exploration of local contexts and social determinants of drowning in Indonesian communities is crucial for ensuring effective water safety and drowning prevention strategies.

**Contribution to Health Promotion**

- This review informs contextualization of drowning prevention and water safety strategies in resource-limited settings of low- and middle-income countries (LMICs).
- This study identified limited community understanding of child drowning preventability, vulnerability and risk factors. Applying the Health Belief Model (HBM), the study revealed a pervasive lack of awareness regarding the susceptibility of children and others in the community to drowning and potential severe consequences of drowning, including injury, disability and death.
- Further investigation of the local contexts and social determinants of drowning, using a health promotion approach, is crucial for effective and sustainable water safety promotion and drowning prevention strategies in Indonesia and other LMICs.

## INTRODUCTION

Despite considerable progress in reducing child mortality from various causes, child drowning persists as a significant yet preventable public health issue worldwide, especially in low- and middle-income countries (LMICs) in the South-East Asia Region, where over 33% of drowning deaths occur (Franklin *et al*., 2020; World Health Organization, 2021; World Health Organization Regional Office for the South-East Asia, 2021). Indonesia, with its vast archipelago of 17,500 islands and population of over 270,000,000, faces particularly high risk, especially among children under 5 years of age (Cenderadewi *et al*., 2024).

Between 2005 and 2019, the under-5 population in Indonesia consistently had the highest drowning mortality rates, averaging 9.67/100,000 population annually (Cenderadewi *et al*., 2024). Children under 5 were 3.67 times (95% CI: 3.63–3.72) more likely to fatally drown than individuals aged 15–49 years, with particularly high risks in Papua, Kalimantan, Sulawesi, Maluku and Nusa Tenggara regions (Cenderadewi *et al*., 2024). Indonesia’s vulnerability to natural disasters such as floods, cyclones, earthquakes and tsunamis, as well as climate change-induced sea-level rise, further compounds the risk (Indonesian National Bureau of Statistics, 2021a; Indonesian National Disaster Management Agency, 2023; Statista, 2023). Despite this heightened risk, there is a lack of understanding about child drowning prevention in Indonesia (Cenderadewi *et al*., 2023). Hence, it is crucial to develop a clear understanding of parental and community perceptions regarding child drowning vulnerability and the associated risk and protective factors to inform drowning prevention and water safety promotion.

The Public Health Model (PHM) is a useful framework that can be used to understand and respond to public health issues, including drowning prevention (Sleet *et al*., 2003; Franklin and Scarr, 2014). The PHM offers a systematic approach, progressing from issue identification to the development, implementation and evaluation of interventions. It emphasizes active community engagement and collaboration with stakeholders to bridge the gap between injury prevention research and health promotion, and its first two stages, of defining the public health problem and risk factor identification, form the foundation of this study (Hanson *et al*., 2012).

There is also a growing recognition of the value of incorporating behavioural science theories into health promotion and injury prevention efforts (Glanz and Bishop, 2010). Given the multifaceted nature of drowning, including its behavioural, natural, physical and social aspects, integrating theories such as the Health Belief Model (HBM) can be beneficial in developing a deeper understanding of perceptions, behaviours and motivations relevant to drowning (Champion and Skinner, 2008; Etheridge *et al*., 2023). The HBM suggests that individuals’ motivation for health-promoting behaviours is influenced by their perceptions of susceptibility and severity of an issue, the perceived benefits and barriers to action, as well as cues to action and perceptions of self-efficacy that influence their likelihood of engaging in health-promoting behaviour (Champion and Skinner, 2008; Etheridge *et al*., 2023). By integrating these constructs, this study seeks to deepen the understanding of community perceptions of child drowning in Indonesia, to inform the development of effective health promotion interventions tailored to local contexts, empowering individuals and communities to adopt water safety-promoting behaviours more effectively.

### Research aims

This qualitative study aimed to explore parental and community perceptions and practices related to perceived causes and risk factors for child drowning in Indonesian communities.

### Research questions

This study answered the following questions:

1. What are Indonesian parental and community perceptions on child drowning as a public health issue in their community?
2. What are Indonesian parental and community perceptions and practices related to causes and risk factors of drowning?

## METHODS

### Study design

This qualitative study is part of a larger mixed-methods study investigating fatal unintentional drowning in Indonesia. The overall study comprised three phases: (i) a scoping review investigating the epidemiology, risk factors and prevention strategies of unintentional drowning in Indonesia (Cenderadewi *et al*., 2023), (ii) a population-based retrospective cohort quantitative study examining mortality rates, incidence rates, risk factors and burden of unintentional drowning in Indonesia between 2005 and 2019 (Cenderadewi *et al*., 2024) and (iii) the qualitative study reported here. The exploratory qualitative design was chosen to expand and explain the findings of the quantitative study, which highlighted high mortality rates among children under 5, particularly in eastern Indonesia (Cenderadewi *et al*., 2024). The qualitative inquiry was grounded in constructivist ontology and interpretivist epistemology, which perceive reality as socially constructed and subjective, acknowledging that participants’ realities are shaped by their social, historical and cultural contexts, emphasizing the uniqueness and subjectivity of their experiences and perceptions (Creswell, 2014; Wright *et al*., 2016). This exploratory qualitative approach was selected for its potential to provide rich insights into participants’ perceptions and experiences of drowning and perceived risk factors for child drowning in their communities, an area with limited exploration in existing literature (Creswell, 2014; Hunter *et al*., 2019).

### Research setting

This study was conducted in seven rural villages in all districts (West Lombok, North Lombok, East Lombok, Central Lombok and Mataram districts) of Lombok Island, West Nusa Tenggara Province of Indonesia, representing coastal and inland communities, and child exposures to beaches, oceans, rivers and other hazards around the household. West Nusa Tenggara was chosen as the study site due to its high under-5 drowning rates of 12.6/100,000 for males and 6.1/100,000 for females in 2019 (Cenderadewi *et al*., 2024) and its predominantly rural character and status as one of Indonesia’s poorest health-performing provinces (Indonesian National Bureau of Statistics, 2021a, 2023a, 2023b, 2024a). The province is characterized by a blend of rural villages, agricultural areas and growing urban centres, with the province’s economy largely reliant on agriculture and fisheries (Indonesian National Bureau of Statistics, 2021b, 2023c, 2024b). While West Nusa Tenggara hosts urban centres with some degree of urbanization and economic activity, a substantial proportion of the province’s population resides in its 1021 rural villages (called ‘desa’ in Indonesia’s administrative hierarchy), in contrast to 141 urban sub-districts (known as ‘kelurahan’, the urban administrative equivalent of ‘desa’) (Indonesian National Bureau of Statistics, 2023d, 2024b).

In 2023, 19% of its population lived below the national poverty threshold, and many barely above it, with key health indicators, such as infant mortality (25 per 1000 live births) and under-5 mortality (29 per 1000 live births), remaining among the nation’s highest in 2022 (Indonesian National Bureau of Statistics, 2023b, 2024a; United Nations Inter-Agency Group for Child Mortality Estimation, 2023). Economic disparities persist, and as reported by the United Nations International Children Emergency Funds (UNICEF) (2019a), only half the population has access to basic sanitation at home, heightening the risk of child drowning near open water bodies. UNICEF also reported that only 20% of 3-year-olds and 40% of 4-year-olds in West Nusa Tenggara are in preschool, whereas preschool attendance is higher in more developed provinces (United Nations International Children Emergency Funds (UNICEF), 2019a, 2019b; Indonesian National Bureau of Statistics, 2023a). For instance, in East Java, where the per capita GDP is nearly 2.5 times that of West Nusa Tenggara, 40% of 3-year-olds and 80% of 4-year-olds attend preschool (United Nations International Children Emergency Funds (UNICEF), 2019a, 2019b). This limited access to early childhood care and education does not align with the World Health Organization’s (WHO) recommendation to promote daycare as a child drowning preventive measure for high-risk under-5 populations (World Health Organization, 2014, 2017, 2021, 2022).

### Sample selection and recruitment

Participants were eligible for inclusion if they were parents of children under the age of 5 or village community leaders, residing in rural villages of densely populated sub-districts located in coastal areas and/or near the inland water bodies of Lombok Island, West Nusa Tenggara Province of Indonesia. Parents were selected due to prior research highlighting their supervisory role as pivotal in preventing child drowning in LMICs (Hossain *et al*., 2015; Cenderadewi *et al*., 2020). Village community leaders, such as village chiefs, elders, religious figures and voluntary community health workers, were chosen for their deep community connections and understanding of local community norms and practices (Rahman *et al*., 2008).

Recruitment employed purposive and snowball sampling, facilitated by village chiefs and community health workers to identify critical informants with substantial insights into the subject. Face-to-face recruitment occurred during social village meetings and activities. Interested individuals were provided with a detailed information sheet and a consent form. Data saturation determined the final sample size of 62 participants.

### Data collection

Between October 2023 and March 2024, seven focus group discussions (FGDs) (*n* = 62) were conducted at various community locations in the local language. This methodology facilitated in-depth data collection, capturing individual narratives and personal experiences regarding child drowning, as well as group-level insights into community norms, practices and decision-making processes related to child drowning prevention. Participants from both groups (parents and village community leaders) were included together in the same FGD. This approach can facilitate discussions where different perspectives interact simultaneously, potentially leading to deeper insights into shared concerns or differing viewpoints. It also allows for the articulation of nuances of the issue and dimensions of social dynamics that might not emerge as readily in separate groups (Hennink, 2014).

Written consent to participate in the study, as well as permission to be audio-recorded, was obtained from each participant. Characteristics such as age, gender, education, occupation, number and ages of children and details about the home environment, including nearby water bodies in the participant’s community and dwelling, were collected via a short questionnaire at the start of each focus group.

A moderator guide was developed by the research team members and informed by the HBM and the findings of the previous scoping review (Champion and Skinner, 2008; Cenderadewi *et al*., 2023). The moderator guide was then translated into Indonesian language. Prior to the implementation, the lead researcher (M.C.) pilot tested the moderator guide for face validity, resulting in minor modifications of the prompts (Table 1).

**Table 1:** Focus group moderator guide’s domains of enquiry and examples of follow-up questions

Domains of enquiry and examples of follow-up questions and probes	Constructs of Health Belief Model applied
Q1. ‘Could you tell me some of the activities your family engaged with around water bodies?’ - Family’s relationship with water: ‘Could you tell me about your family’s and children’s activities around water?’ ‘Could you tell me what water bodies exist in your community and in and around your home?’ ‘Could you tell me your use of watercrafts and flotation devices on board?’ - Supervision for children: ‘Could you tell me more about your family structure and the main caretaker in your household?’ ‘Could you tell me who in your family is responsible to supervise children while they are doing activities around water?’	Perceived susceptibility
Q2. ‘What do you think are the greatest health concerns for your community?’ - ‘How important do you think drowning is as a health issue in your community?’ - ‘Where do you think drowning fits among these greatest health concerns in your community?’ - ‘Have you ever experienced/witnessed/heard stories about drowning events in your community?’ - ‘Are you aware of local beliefs and practices surrounding the issue of drowning in your community?’	Perceived susceptibility Perceived severity Cues to action
Q3. ‘Who do you think is at most risk for drowning in your community?’ Q4. ‘What do you think of drowning as a cause of injury/death for children?’ Q5. ‘What do you think are the reasons that might cause a child to drown?’ Q6. ‘Can you tell me about aspects of the environment and community in which you live that could increase the risk of a child drowning?’	Perceived susceptibility Perceived severity Perceived barriers Self-efficacy Cues to action
Q7. ‘What are some of the things that might make it hard to keep children safe from drowning?’ Q8. ‘Can you tell me about how and what have you taught your children about water dangers?’ Q9. ‘What would you like to see put in place to prevent children from drowning in your community?’ - ‘Who do you think are responsible for preventing drowning?’ - ‘Where do you get your information on drowning prevention from?’	Perceived barriers Perceived benefits Self-efficacy Cues to action

Two female Indonesian researchers (M.C. and P.B.F.) facilitated the focus groups, with one (M.C.) facilitating discussions and the other (P.B.F.) taking notes. Both were fluent in Indonesian, Sasak and English. Discussions were primarily in Indonesian, but participants could also use the local Sasak language. Each group lasted 50–60 min and consisted of 5–12 participants.

Discussions were audiotaped and transcribed verbatim. The Indonesian transcripts were translated into English by the lead researcher (M.C.) and then back into Indonesian by another team member (P.B.F.) to ensure accuracy. Senior researchers (S.G.D. and R.C.F.) reviewed translated transcripts to verify data accuracy.

### Analysis

Translated transcripts, demographic data and field notes were entered into NVivo Version 20. This study employed thematic analysis (TA), guided by Braun and Clarke’s framework (Braun and Clarke, 2006), for its suitability in exploring child drowning in Indonesia, offering a flexible method to investigate a range of research questions in this under-explored area. While this study acknowledged the theoretical grounding provided by the HBM (Champion and Skinner, 2008) and integrated it into a deductive approach to analysis, the analysis favoured open and organic coding. Themes emerged through iterative data immersion, reflection and questioning, underscoring the subjective and interpretative nature of TA. Thus, researcher reflexivity is crucial, rather than emphasizing objectivity, reliability, or coding accuracy, aligning with reflexive TA principles which allow for flexibility and adaptability (Braun and Clarke, 2006, 2021, 2023). This approach allowed for in-depth exploration and interpretation, uncovering rich insights into the complexity of child drowning in rural Indonesian communities.

The TA progressed through several stages (Braun and Clarke, 2006, 2023). First, data immersion involved the researchers thoroughly familiarizing themselves with the dataset by repeatedly reading focus group transcripts. Second, code development entailed generating initial codes across the dataset by systematically labelling segments of data representing meaningful concepts, ideas and patterns. The researchers started with deductive coding based on the theoretical framework of the HBM to guide the initial stages of analysis. However, as analysis progressed, new sights emerged organically beyond the original framework. This approach allowed for a nuanced exploration of both theoretical expectations and unexpected findings within the data, reflecting a balance between deductive and inductive approaches. Thirdly, relevant codes were grouped together into potential themes.

The next step was iterative organizing, structuring and refining of data to capture key concepts and phenomena within the data set. This was followed by clearly defining each theme, setting its scope and boundaries, developing its narrative and giving it an informative name, ensuring that each theme was distinct and coherent. The researchers then moved beyond descriptive analysis to interpret the meaning and significance of the themes within the research context, exploring relationships between themes, considering how specific social, cultural and historical contexts shaped meanings, identifying underlying assumptions or theoretical frameworks and considering the broader implications of the findings. The findings were subsequently organized and presented in a thematic table (Table 3), which provided detailed theme definitions and scopes and representative excerpts of each theme. The researchers then developed narratives for the four identified themes, integrating the data and providing the contextual basis, as presented in the *Results* section of this publication (Braun and Clarke, 2006, 2023).

**Table 3:** Definitions of themes and illustrative quotes supporting interpretations of themes

Themes	Definition and inclusions	Sub-themes	Representative excerpts
Concepts of drowning as an issue	Participants’ views and concepts of drowning as a public health problem in their community, and of the severity of drowning consequences.	Perceptions of drowning susceptibility	*‘We never worried about drowning. We don’t have people drown around here. Where would they drown? We don’t have big rivers or lakes. Maybe for those closer by the beac*h’. (Group 2, female, participant ID number: G2F1) *‘Maybe it’s fishermen who are more at risk for drowning. People who go to the sea every day’.* (Group 7, female, participant ID number: G7F2)
		Perceptions of severity of drowning consequences	*‘I never thought about drowning like that [as a cause of injury/death in children].’* (Group 1, male, participant ID number: G1M1) *‘Children could be ill or die from drowning’.* (Group 2, female, participant ID number: G2F5) *‘Yes, death by drowning in children is possible. They could die from swallowing too much water’.* (Group 3, female, participant ID number: G3F5)
		Personal experiences shaped perceptions on children’s susceptibility to drown	*‘I entered the bathroom, just by pure coincidence, then I saw the child was already in the bathtub, flailing. The child was not conscious. He was limp. I was so scared. … That’s why we need to supervise them closely’.* (Group 6, male, participant ID number: G6M1, rescued his nephew from drowning) *‘[Name redacted]’s child almost drowned once. In the bathtub. He put his head into the water in the tub. Yes, it could happen around us, especially with young children. And particularly when the tub is large, and the child is little. Especially in the bathroom, and with children as young as around 5 years old. You need to accompany your children when they are in the bathroom. … Experience is a lesson’.* (Group 6, female, participant ID number: G6F1, witnessed a non-fatal drowning incident of her neighbour’s son)
Perceived preventability of child drowning	Participants’ views that child drowning is an inevitable accidental event, including underlying local cultural and religious beliefs that drowning is destined by God or caused by the presence of spirits.	Drowning is an inevitable event	*‘Drowning is destiny. It is a tragedy, an accident’.* (Group 1, female, participant ID number: G1F5) *‘Drowning happens because the child slips and falls into the water’.* (Group 3, female, participant ID number: G3F2) *‘Drowning is a matter of death. Death comes from God. Death from drowning is usual. It’s destiny. When death by drowning is destined for them, it’s their fate, as promised by God’.* (Group 1, male, participant ID number: G1M1) *‘This person, an adult, as old as me perhaps, drowned in a waterfall … The person drowned within minutes and cannot be found in those minutes. Perhaps the water has spirits, spirits that wait on it’.* (Group 5, female, participant ID number: G5F3)
	Participants’ views that child drowning is a preventable event, including their logical reasonings behind their practices in reducing the risk of drowning.	Drowning is a preventable event	*‘[Child drowning happens] because children are curious. Even if they’re in the swimming pools with their parents, for example, pools have different level of depths, and children can just run and plunge into the deeper pool. So as parents you need to tell them what to do beforehand’.* (Group 4, female, participant ID number: G4F3) *‘Drowning happens because children will go with many friends, but they’re all children. If anyone drowns, they will help one another and they will drown, since none of them can swim’.* (Group 2, female, participant ID number: G2F1)
Drowning risk factors	Demographic, behavioural, cultural and environmental characteristics that participants associated with a higher likelihood of drowning event taking place.	Age Young children	*‘Children are the most prone to drown’.* (Group 5, female, participant ID number: G5F2) *‘That’s why we are concerned about children going out by themselves, such as to the beach, for we have small children’.* (Group 4, female, participant ID number: G4F3)
		Adolescent and young adults	*‘Teenagers, three teenagers died from drowning’.* (Group 6, female, participant ID number: G6F1) *‘In East Lombok, here, some time ago, university students, while undertaking a community project, had their legs pulled in by the water. Three university students. They all died’.* (Group 5, female, participant ID number: G5F6)
		Gender	*‘Especially boys [are prone to drowning]. It is very difficult to tell boys what to do. They tend not to listen to you’.* (Group 2, female, participant ID number: G2F4) *‘Oh no, boys are not more at risk to drown. My granddaughters are much braver than the boys. They are very brave in playing with waves on the beach’.* (Group 3, female, participant ID number: G3F5)
		Locations Natural environments	*‘We heard many [drowning] cases these days from the news, especially on the beach’.* (Group 6, female, participant ID number: G6F1) *‘Drowning occurs to people who are on board of boats, in the sea’.* (Group 3, female, participant ID number: G3F1)
		Man-modified bathing pools/ponds	*‘I tell my kids not to go the public bathing ponds. I tell them you might drown there’.* (Group 2, female, participant ID number: G2F1) *‘Perhaps there will be drowning in places like Narmada [a subdistrict in West Lombok, known for its water springs, public swimming pools, and bathing ponds, serves as popular public destinations], where there are plenty of water-related destinations. In bathing pools and ponds there’.* (Group 5, female, participant ID number: G5F4)
		Home environments	*‘Drowning can happen in buckets. People these days have big, tall buckets. So, it’s dangerous, when bathtubs and buckets are not covered. But the thing is, because we often use the tubs and the buckets, that, even if they are covered, we will uncover and cover them back and forth because we take water from them multiple times a day. And children can open them themselves too … The bathtubs here are big sometimes, so children can fit in easily. My child can plunge all into it!’* (Group 5, female, participant ID number: G5F1) *‘Perhaps children could drown when wells are not covered’.* (Group 5, female, participant ID number: G5F2)
		Environmental factors Lack of barriers to water bodies	*‘We truly stress it to them to avoid playing around uncovered wells, to avoid them falling over the edge of the wells’.* (Group 2, female, participant ID number: G2F1) *‘The ditches in this village are not covered. Ditches perhaps can be covered [to reduce the risk of drowning]’.* (Group 3, female, participant ID number: G3F2)
		Weather conditions	*‘I told my children that when the volume of water on the stream is high, it’s better for you not to go there. I often scare them, telling them that you could drown and there will be no one to save you, so that they will not play there. Especially during rainy season’.* (Group 2, female, participant ID number: G2F4) *‘On ferries, lifejackets are being stored in one area. I observe the weather, usually. If the weather looks bad, I will sit near where lifejackets are stored … Everyone can drown, depending on the situation. It depends on the weather’.* (Group 6, male, participant ID number: G6M1)
		Disasters	*‘No drowning in this community. We don’t have floodings around here, maybe on the coast, near the sea … No drowning. It’s drier these days. It never floods in this area’.* (Group 1, male, participant ID number: G1M1) *‘Never heard of drowning, but we have experienced earthquakes, when they said there will be tsunami’.* (Group 2, female, participant ID number: G2F1)
		Behavioural factors Lifejackets use	*‘No lifejackets [on board public boats], which irked me, especially because the tides were so strong. There were lifejackets on the top deck, but we were not given them to wear. No one gave instructions or warned the passengers to wear lifejackets … I observe the weather, usually. If the weather looks bad, I’ll sit near where lifejackets are stored’.* (Group 6, male, participant ID number: G6M1) *‘No, the boat crews never do that [informing passengers where the lifejackets are stored]. We took public boat sometimes, going out from the island, with kids. No one uses lifejackets. I don’t know if it’s available’.* (Group 2, male, participant ID number: G2M2)
		Children’s behaviours	*‘Drowning happens if the children are naughty. Some of the kids are too naughty. They know that the pond is deep, but they are still willing to take the risk’.* (Group 2, female, participant ID number: G2F2) *‘We give them a lot of advice, not to play when the water rises. But kids are stubborn. They will always go on their own way with their friends’.* (Group 5, female, participant ID number: G5F2) *‘Maybe children being playful with each other causes drowning. Their friends being playful while playing. Young children probably don’t understand anything about the danger in water’.* (Group 5, female, participant ID number: G5F1)
Implications of cultural norms and collectivism in childrearing practice on drowning risk	Participants’ views and practices on childrearing, including the influence of local cultural and gender norms, as well as the involvement of family and community members in childrearing and supervision practices.	Childrearing practices in the local community	*‘It’s different with fathers. Fathers leave for work every day, while the responsibility to keep children safe at home is with the mothers’.* (Group 2, female, participant ID number: G2F1) *‘For me, as a homemaker, it is difficult for me to keep an eye on my children. Here, in this community, usually parents have, like, a spare parent, the grandmother, who often act as a caretaker, the second caretaker of the children’.* (Group 7, female, participant ID number: G7F2) *‘There are always many community members doing their activities around, so they can look after the children from any harm’.* (Group 2, female, participant ID number: G2F4) *‘I told my older children, to take care of their younger siblings, so they will not drown. And to not take their younger siblings to the beach, for fear of them being dragged away by the waves’.* (Group 1, female, participant ID number: G1F2)
		Supervision while children play around water bodies	*‘Yes, it’s common here, mothers letting their kids playing on their own. Children often play around streams too. And many older children go play further from here, to ponds around here. … Many children are not accompanied by their parents while playing around water’.* (Group 4, female, participant ID number: G4F1) *‘Yes, it’s common for children here to play unsupervised. Mothers often let children play among themselves, because the mothers are probably busy at home as well’.* (Group 4, female, participant ID number: G4F3)

### Researcher reflexivity on positionality

This study acknowledges the positionality of two Indonesian researchers (M.C. and P.B.F.) as middle-class, well-educated medical doctors, alongside the senior researchers’ (R.C.F. and S.G.D.) non-Indonesian backgrounds. Both Indonesian researchers are from Lombok, fluent in Sasak and Indonesian, offering a unique connection to the region. Throughout the analysis, the researchers consistently reflected on and critically examined how their socioeconomic positions, privileges and assumptions, as well as the power dynamics with the underserved populations studied, might introduce biases and they explored strategies to mitigate them. Advice from community leaders and prolonged engagement at the study site deepened the researchers’ understanding of the research setting and participants, centring community voices and ensuring an authentic representation of participants’ perspectives.

### Rigor

To ensure the dependability, credibility, conformability and transferability of the study, several measures were taken: (i) purposive sampling; (ii) prolonged engagement at research sites to build rapport with participants and to gain a full understanding of participants’ narratives, (iii) collection of participant demographics; (iv) continual reflection on assumptions and biases; (v) member-checking, conducted both informally during each FGD to immediately verify findings, and formally through follow-up interviews after FGD transcriptions, to validate the interpretation of the data collected; (vi) back-translation to validate data accuracy; (vii) iterative cycles of coding, interpreting and reflecting on data; (viii) consensus discussions on themes and theme definitions; (ix) inclusion of direct quotations as evidence and (x) establishment of an audit trail of data collection and analysis (Raskind *et al*., 2019; Braun and Clarke, 2021).

## RESULTS

Sixty-two parents of children under-5 years of age and village community leaders participated in the focus groups. The majority were female (*n* = 47, 75.8%), aged between 25 and 44 (*n* = 32, 51.6%), and had not completed primary education (*n* = 36, 58.1%). Most participants recruited were mothers of children under the age of 5 (*n* = 33, 53.2%), followed by village community leaders (*n* = 23, 37.1%) and fathers (*n* = 6, 9.7%) (Table 2). The village community leaders were predominantly parents of children aged over 5 years or grandparents of children under 5. This demographic composition provided a deeper understanding of the community context surrounding child drowning.

**Table 2:** Focus group participants’ characteristics and home environment

Focus group participants’ characteristics and home environment (*n* = 62)			
		*n*	%
Gender	Female	47	75.8
	Male	15	24.2
Type of participant	Mothers	33	53.2
	Fathers	6	9.7
	Village community leaders	23	37.1
Age group	18–24 years	6	9.7
	25–34 years	16	25.8
	35–45 years	16	25.8
	44–54 years	11	17.7
	55 and above	13	21.0
Education level	Did not complete primary education	36	58.1
	Completed primary education	14	22.6
	Completed high school	12	19.4
Occupation	Homemaker	34	54.8
	Daily labourer	7	11.3
	Farmer	4	6.5
	Fisherman	2	3.2
	Others	15	24.2
Number of children	0	1	1.6
	1	10	16.1
	2	11	17.7
	3	16	25.8
	4	12	19.4
	5 and over	12	19.4
Number of children under the age of 5	0	23	37.1
	1	28	45.2
	2	11	17.7
Water bodies exist within 500 metres from participant’s home	Wells	62	100.0
	Ditches	46	74.2
	Creeks	46	74.2
	Rivers/larger streams	13	21.0
	Beaches	12	19.4
	Ponds	1	1.6
Water bodies exist in and around participant’s home	Wells	60	96.8
	Bathtubs (serves as water containers)	50	80.7
	Bucket	55	88.7

Data analysis revealed four key themes: (i) concepts of drowning as an issue; (ii) perceived preventability of child drowning; (iii) drowning risk factors; and (iv) implications of cultural norms and collectivism in childrearing practice on drowning risk. Results are presented below under each theme heading and summarized in Table 3, which includes further illustrative participant quotes.

### Theme 1—Concepts of drowning as an issue

Most participants across all age groups, particularly village community leaders, did not perceive drowning as a risk in their community. Those who did view drowning as a possibility, mostly saw it as an issue for those who lived near a beach. Only a few participants acknowledged drowning as a health concern in the community, mostly those who had personal experience of a non-fatal child drowning incident in their home or community.

*There is no such thing [as children drowning]. Maybe it’s them who live by the beach. Perhaps they are the ones who should pay more attention and supervise their children. It (drowning in children) seems like too far away of an idea.*

Group 3, female, participant ID number: G3F1

*Actually, at times we are worried about it [drowning], especially when the kids are playing in the streams. That is why we look for them when they’ve been gone playing for too long.*

Group 2, female, participant ID number: G2F4

Overwhelmingly, participants had considered drowning as a risk for adults working on the open seas. However, they had not considered the susceptibility of children drowning and showed limited awareness of the possibility of children drowning in and around the home environment.

*I think maybe it [drowning] is for people who work at the sea. Boaters, fishermen go to the sea every day. Drowning depends on the location, really. If it’s in and around the home, where would these children drown?*

Group 2, male, participant ID number: G2M2

While some participants recognized that drowning could be serious and cause death, many, particularly those who appeared to be in older age groups, downplayed its severity. Most participants cited other illnesses such as fever, respiratory tract symptoms and diarrhoea as the primary health concerns for children in their community, with little consideration of drowning.

*No, not death [by drowning]. No. Maybe [drowning causes] like regular runny nose, from inhaling the water.*

Group 1, female, participant ID number: G1F3

*The most important health problems here are usually related to children’s illnesses. Fever, coughs, shortness of breath. But these kinds of illnesses are common in this village.*

Group 5, female, participant ID number: G5F2

Some participants who appeared to be in younger age groups were more likely to recognize the severe consequences and potential fatality of drowning. Many of these participants cited acquiring awareness of the fatal consequences of drowning, including in children, from various media outlets, including news broadcasts on television and social media, emphasizing the importance of preventive measures, particularly parental supervision of children.

*Drowning is important, and it is fatal because it can cause death from lack of oxygen. Children can pass out from it. It is a serious matter. 50:50 chance of survival, between life or death.*

Group 6, male, participant ID number: G6M1

*There are more news reports on drowning deaths these days, including those involving celebrities. If any celebrity or their children drown, we become more aware. The news report becomes a lesson for us that we need to be more cautious. We need to be careful not to trust other people too easily, including in letting our children be supervised by others.*

Group 6, female, participant ID number: G6F1

Interestingly, none of the participants mentioned how non-fatal drowning can cause injuries or disabilities. While some participants acknowledged the consequences of non-fatal drowning, they primarily referred to it as an ‘illness’ resulting from drowning, with no mention of injuries or disabilities caused by drowning.

*Not only can drowning cause an illness in children, but drowning can also cause children to die.*

Group 4, female, participant ID number: G4F1

*Drowning can cause illness from wrongly inhaling the water.*

Group 1, female, participant ID number: G1F2

Some participants reported that witnessing non-fatal child drowning incidents in their home and community changed their perception of children’s susceptibility to drowning and the potential severity of its consequences. These incidents also heightened their awareness of the importance of providing constant supervision for children.

*My child almost drowned. My youngest child. Four and a half years old… My child almost didn’t have the strength to survive, because he is still very young. I was the one who was being careless. I asked my son’s friend to watch over him, but he didn’t pay attention. I left both of them to play, thinking they would be fine. I went inside. The water level was very shallow, so I didn’t think my son could drown in it, but apparently, children can still drown in shallow waters. It [the experience] must have changed my opinion [on child drowning risk]. Accidents could happen and cause deaths.*

Group 6, female, participant ID number: G6F3

### Theme 2—Perceived preventability of child drowning

Many participants viewed child drowning as ‘accidents’. They perceived drowning incidents as unexpected and inevitable occurrences rather than preventable events that can be mitigated through appropriate measures. Some participants also described drowning as an unintentional event.

*I think drowning is similar to road traffic accidents. It is an accident caused by carelessness.*

Group 6, male, participant ID number: G6M1

*Children drown if they slip. Slip and fall into the water.*

Group 1, female, participant ID number: G1F2

Participants’ perception of the inevitability of child drowning incidents is influenced by deeply rooted cultural and religious-based fatalistic beliefs surrounding drowning. Many participants cited local cultural and religious beliefs, attributing drowning to destiny or supernatural causes such as ‘spirits’.

*In our culture, in Sasak culture, death is our destiny, the end of life. It is a promise already made by God, determining how our lives will end. If drowning is our destiny, we must accept it.*

Group 4, female, participant ID number: G4F1

*Well, there are local beliefs. It’s, I don’t know how to explain it because these things cannot be seen with our eyes, but the water spirits are believed to drag people in. Logically, perhaps, if we would like to rationalise, there could be a whirlpool in the water that drags people in. But people still believe in such things. There are sacred places and sacred waterways believed to cause people to drown.*

Group 6, male, participant ID number: G6M1

However, some participants, despite believing drowning is inevitable due to destiny, still recognized the importance of taking preventive measures, especially for their children. They outlined specific safety skills children need to be equipped with, particularly swimming ability, and safety approaches parents would adopt to reduce the risk of their children drowning, such as providing supervision and attempting to access lifejackets on public boats, even though lifejackets are often not provided to passengers.

*If the boat goes down, you’re going to drown. That’s it. If I’m on my own, yes, I can say it [drowning] is my destiny, I can accept that. But if I’m with my kids, I don’t think I can accept that [drowning] that easily, that I’m going to drown with my children. That’s why I’m looking for a safer spot on the ferry … if the winds were strong, we told our children to sit under the lifejackets, near where the lifejackets were hanged, as if we could quickly grab them [lifejackets].*

Group 4, female, participant ID number: G4F3

*Child drowning happens because no children here know how to swim.*

Group 5, female, participant ID number: G5F6

### Theme 3—Drowning risk factors

Participants identified a range of risk factors that make children vulnerable to drowning including age and gender of the child, behavioural practices and exposure to water hazards in the wider home, community and natural environments.

The age of children was discussed as a risk for drowning with younger children being perceived as being particularly vulnerable. Some participants reported incidents of younger children drowning in public aquatic environments such as beaches. Several others recognized that children are prone to drowning but believe that such incidents will occur outside of their community.

*There [on the beach], we often heard people drowned, especially little children.*

Group 4, female, participant ID number: G4F1

*Young kids are prone to drown. Kids from other villages. Not here.*

Group 1, female, participant ID number: G1F7

Others viewed adolescents or young adults to be at particular risk, basing their views on past incidents observed in their communities and reported on the news, including on social media. Some participants attributed the heightened risk in adolescents to the difficulty of supervising them, their newfound independence and their tendency to engage in unsafe water environments influenced by peers.

*There were drowning cases in Bima, I heard. There were several cases of drowning there, where young men drowned in the streams. Drowning is also commonly heard where I’m from, in my village. Also, many drowning cases here in Lombok. I heard it some time ago on tv and on social media, of a young man drowned and being carried by the stream up to Ampenan [a coastal sub-district in Mataram, Lombok Island].*

Group 5, female, participant ID number: G5F6

*When our children were younger, they only played around the house. But once they grew bigger, it’s a different level of delinquency, isn’t it? They can travel outside of the village by themselves. They can go to the bathing ponds by themselves*

Group 2, female, participant ID number: G2F1

Participants had differing views regarding whether gender was a risk factor for drowning. Some suggested boys were more prone to being ‘naughty’ and disobey parents’ advice to stay away from open or unsafe water bodies, hence more likely to drown, while others saw no difference in drowning risk between boys and girls and perceived that drowning could result from ‘naughty’ behaviour in both.

*I think boys and girls these days are the same regarding the risk of drowning. Both can be naughty.*

Group 4, female, participant ID number: G4F1

Participants discussed various environments posing drowning risks for children, including natural, built and home settings. Most cited natural aquatic locations, emphasizing beaches and open seas as the main locations of concern, while some also mentioned larger rivers and lakes.

*In general, everyone has the risk to drown on the beach, with big waves, and people who cannot swim. I once helped rescue a female who was drowning on the beach … I helped drag her out of the water.*

Group 6, male, participant ID number: G6M1

**Fig. 1:**
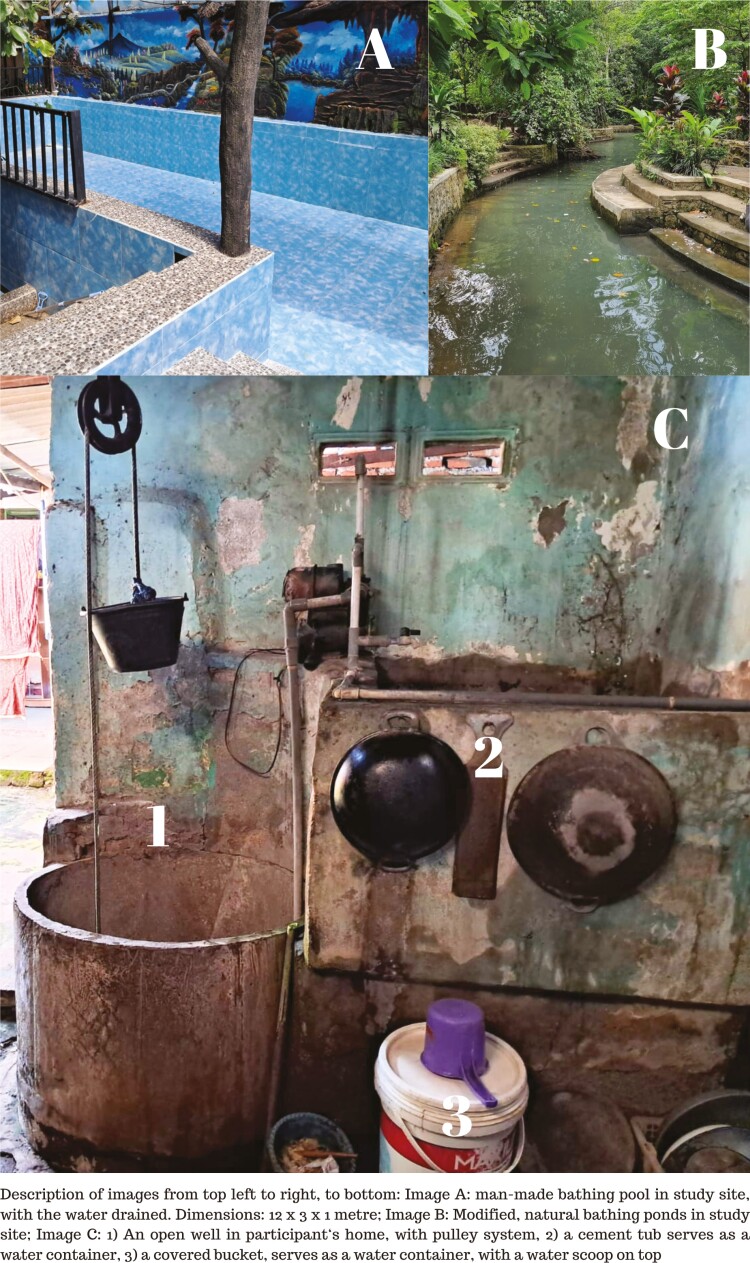
Examples of water bodies in study site.

Participants recognized bathing pools/ponds as potential drowning sites. Participants described that bathing pools/ponds can be either manmade structures resembling swimming pools, albeit shallower and smaller, or modified natural water sources with embankments and coping built around them (Figure 1). Locals, especially in rural areas, often lack swimming skills, hence referring to these facilities as ‘bathing pools’ or ‘bathing ponds’ for dipping and cooling off rather than swimming.

Many participants noted children often went to bathing pools without parental or lifeguard supervision, which heightened their risk of drowning. Some mentioned that the low entrance fees of privately owned bathing pools make them accessible to children. Participants also noted that these pools do not require parental attendance or impose minimum age limits for children.

*The children went there [to public bathing pools] with their friends. So, I think they could drown without the parents knowing. We call it “Kolam dua ribu” [in English: ten US$ cents bathing pool, referring to the entrance fee]. Some of the pools are quite deep, and these kids can plunge into the deeper pool, and nobody will know.*

Group 2, female, participant ID number: G2F5

*Children can enter these bathing ponds without parents, any time they wish.*

Group 4, female, participant ID number: G4F3

Several participants, particularly those who appeared to be younger parents, recognized the risk of child drowning at home. They identified ‘tubs’ or ‘bathtubs’, common as water containers inside Indonesian bathrooms, along with buckets and uncovered wells (example provided in Figure 1), as potential drowning sites in and around home. Incidents of non-fatal drowning in these household water sources were also cited by some participants. They mentioned that the drownings occurred when they were not supervising their children while playing around water in and around their homes.

*My son was also once played in the bathtub and almost drowned! I wasn’t aware of that because he was playing in the bathroom. People here don’t close their doors, their bathroom doors, so children can walk in and out … I heard the sound; “Gulp. Gulp.” It was my son! I wasn’t aware of him drowning. I thought, where is my child? The next thing I saw was his head was in the tub! He was still little at that time. Five years old.*

Group 5, female, participant ID number: G5F1

Unprotected wells and ditches in communities were also perceived as environmental risk factors of child drowning. Some participants mentioned that they had covered their wells to prevent children from falling in. However, they noted that some community members still maintain open wells in their houses.

*Yes, kids were curious about the uncovered well and what was inside, so they got closer to it and peeked in. We were worried about the possibility of children falling into the well, so most of us have covered our wells.*

Group 2, female, participant ID number: G2F1

*But no one worries about the possibility of falling into wells. If there are any children playing around the well, we just need to yell at them to stop playing around the well.*

Group 2, male, participant ID number: G2M1, owns an open well in his house

Participants identified various weather conditions as environmental risk factors for drowning, particularly during the rainy season. They also highlighted the dangers posed by powerful waves, high tides and strong offshore winds, which increase the risk of drowning both in natural water bodies and during water transport-related incidents.

*I tell them [the children] not to go around water during the rainy season, because the water will rise, and we might get carried away by the water flow.*

Group 7, female, participant ID number: G7F1

In addition to rainy season flooding, most participants, whether coastal or inland residents, voiced their concerns about earthquakes and the risk of tsunamis. They referenced the 2018 series of 6.9 magnitude earthquakes in northern Lombok, which triggered small-scale tsunamis on the northern coast of the island, as a significant event that heightened their awareness of their susceptibility to tsunamis and the associated risk of disaster-related drowning.

*We were afraid of the seawater! Nowhere felt safe! … We’re not afraid of floodings, but we’re very afraid of tsunamis!*

Group 2, female, participant ID number: G2F4

While several participants viewed lifejackets as an important safety device, most stated that lifejackets were rarely available or used on public boats. This lack of access to lifejackets was seen as a factor contributing to their vulnerability to drowning in watercraft-related incidents.

*No one on board of boats use lifejackets. Never. The boat crews never let people know that we must use lifejackets and where the lifejackets are stored. And I have taken a few boat and ferry rides, to Bali and to Java, but no such information on lifejackets. Sometimes there were lifejackets around, sometimes no lifejackets can be seen around. But no there was no information on that [lifejackets], especially on smaller boats.*

Group 2, male, participant ID number: G2M1

Many participants identified children’s behaviours as drowning risks. Some perceived children’s behaviours as ‘naughty’ which led them to being in risky situations. Meanwhile, some participants who appeared to be younger parents, attributed the risky behaviour to children being naturally curious and active as part of their developmental phase.

*Mostly, it depends on the child’s bravery. Even though the parents have watched their children, but children can be stubborn.*

Group 5, female, participant ID number: G5F3

*Especially children around the age of 5 years old. That’s why we need to supervise them closely. They’re very active around this age. We need to watch them, what are they playing, how they are playing.*

Group 6, male, participant ID number: G6M1

### Theme 4—Implications of cultural norms and collectivism in childrearing practice on drowning risk

Participants highlighted the crucial role of supervision in preventing child drowning and discussed cultural norms and practices related to childrearing. Predominantly, participants across different age groups and both genders noted the cultural significance of mothers in childrearing. Participants described traditional gender roles in childcare and household duties, where mothers were primarily responsible for child safety alongside household chores, while fathers were seen as providers. Many mothers expressed challenges in balancing these responsibilities.

*The younger kids are usually with the mothers. While we hand-wash the clothes, with the water from the well in the buckets, our kids also play around the well and buckets. They’re with us. We tell them not to play far near the streams or ditches. We’re afraid that they may slip and fall into the water.*

Group 2, female, participant ID number: G2F2

*As a stay-at-home mother, who must do everything by myself, yes, it is particularly difficult to balance between completing house tasks and taking care of my children. But it’s different for men. It seems as if men only need to earn money and that’s it. Meanwhile, women must wash, cook, clean the house, care for the children, some also work—everything.*

Group 5, female, participant ID number: G5F6

Most participants also highlighted the cultural tradition of involving extended family, including in-laws, grandmothers and older children, and neighbours in caring for young children. Mothers are often unable to provide constant supervision for their children, leading them to rely on their older children or children in the neighbourhood to supervise their children while playing, including around water bodies. These practices could either protect children from drowning or heighten their vulnerability.

*Children can be watched by in-laws. Or helped by their grandmothers, usually. Many members in this community play an important role in caretaking children. The grandparents, aunts, neighbours. We could watch each other’s children.*

Group 6, female, participant ID number: G6F1

*Children will watch themselves. It is common here in the village. They’ll take care of each other.*

Group 7, female, participant ID number: G7F1

## DISCUSSION

Child drowning is a significant public health issue in Indonesia. As highlighted in the first and second stages of the PHM, it is important to understand current parental and community perceptions and practices related to child drowning, to better comprehend how child drowning is perceived as a public health problem in the community as well as its perceived causes and risk factors, to inform future health promotion approaches to address the issue (Sleet *et al*., 2003; Hanson *et al*., 2012). This study has yielded valuable insights into the community’s views on drowning as a cause of childhood mortality and injury, as well as identifying contextual factors that put children at risk of drowning in Indonesia.

Despite evidence of child drowning as a leading cause of death among Indonesian children (Cenderadewi *et al*., 2024), the study identified a limited understanding regarding children’s vulnerability to drowning, its preventability and associated risk factors. Utilizing constructs from the HBM, this study identified that most participants were unaware of the susceptibility of their children and others in the community to drowning, and the severe outcomes that can result such as injury, disability and death, highlighting the urgent need to enhance public awareness about drowning.

The study explored HBM constructs to identify perceived risk factors contributing to drowning, including being young children, adolescents and young adult males, behavioural practices such as inadequate supervision and non-usage of life jackets, and broader environmental factors within homes, communities and natural settings. HBM has been widely recognized and applied in various studies to better understand individual attitudes and behaviours towards drowning risks and related preventive behaviours (Mel *et al*., 2017; Glassman *et al*., 2018; Abercromby *et al*., 2021; Willcox-Pidgeon *et al*., 2021; Tengecha *et al*., 2022). For instance, research by Mel *et al*. (2017) applied the HBM to assess parental and caretakers’ perceptions regarding a mass media messaging campaign on child drowning risk. Their study highlighted how the model serves to identify the campaign’s effectiveness in communicating children’s susceptibility to drowning and the presence of water hazards in and around the home (Mel *et al*., 2017). Another study by Abercromby *et al*. (2021) utilized the HBM to explore young adults’ beliefs and behaviours related to alcohol consumption and water safety, revealing the role of peers, social influences and cultural norms in their decision-making regarding alcohol consumption during water-based activities through the exploration of HBM constructs.

Participants generally associated drowning with beaches or open seas, despite evidence indicating that child drowning incidents often occur in smaller water bodies such as unprotected wells, canals, ponds and streams, similar to those that exist in participants’ communities (Tyler *et al*., 2017; Zaara *et al*., 2022). Direct exposure to child drowning incidents prompted some awareness on the likelihood of such occurrences in and around homes, with participants identifying open wells, tubs and buckets as notable risk factors in and around homes, consistent with previous research in other LMICs (Morris *et al*., 2016; Tyler *et al*., 2017; Zaara *et al*., 2022; Siddiqui *et al*., 2023). Participants highlighted that they acquired awareness of child drowning risks and consequences through media, particularly social media. This underscores the need for enhanced strategic communication, aligning with WHO recommendations, via media outlets, including social media, with parents being a key target audience, to bolster public awareness on child drowning risks and prevention, and to support effective implementation of prevention strategies (World Health Organization, 2017).

Due to most participants not having previously considered child drowning as a significant issue or one that potentially has serious consequences, there was little discussion about intent to change behaviour or practices to prevent drowning (Glanz and Bishop, 2010). While direct exposure to drowning incidents prompted some awareness of the issue, most participants were in the early stages of behavioural change regarding protective strategies (Kowalski *et al*., 2014). Although supervision was acknowledged as crucial to prevent child drowning, Indonesian mothers found it challenging to supervise their children constantly and often delegated supervision to other family and community members, despite previous research suggesting that this practice may be inadequate to prevent child drowning (Blum *et al*., 2009; Morrongiello *et al*., 2010; Peden and Franklin, 2018). The failure to perceive children being susceptible to drowning and lack of acknowledgement of the potential severe consequences of a drowning incident could stem from cultural norms accepting drowning occurrences as inherently inevitable and part of destiny. This perception of drowning as predestined by fate is similar to what has been reported in Bangladesh (Blum *et al*., 2009) and India (Lukaszyk *et al*., 2019). This view of the inevitability of drowning is further compounded by inadequate resources and infrastructure at both the home and community levels. Therefore, further exploration of the local context and social determinants of drowning across Indonesian communities is essential to ensure that proposed health promotion approaches are effective and feasible.

The study also highlighted a gap in understanding awareness and attitudes towards child drowning risks among various community segments, emphasizing the necessity for separate investigations into parents and community leaders. These studies would inform targeted strategies tailored to each group. To develop effective strategies aligned with local contexts, beliefs and practices, a collaborative co-design approach involving researchers, stakeholders and community members is recommended (Clark *et al*., 2022; Koon *et al*., 2023; Peden *et al*., 2023). Previous research, such as in Australia, has demonstrated that co-design effectively customises drowning prevention interventions to meet local needs, bolstering community support and ensuring their long-term sustainability (Koon *et al*., 2023). Further investigation is needed to assess its applicability in designing culturally sensitive and context-specific interventions suitable for different community segments in Indonesia.

While this study provides useful information that enhances our understanding of how parents and community members feel about child drowning as an issue and what they see as risk factors, further research into comprehensive health promotion strategies, grounded in a socio-ecological approach, is imperative to achieve effective and sustainable drowning prevention efforts in Indonesia. This involves examining the interconnectedness of drowning prevention with initiatives such as provision of safe aquatic environments, infrastructure investments, affordable childcare and early childhood education, equitable education, rural development, enforcement of boating and maritime safety regulations, disaster risk management and efforts to alleviate socioeconomic disparities. This investigation is particularly crucial for socioeconomically disadvantaged populations in rural and remote regions across Indonesia’s archipelago.

### Strengths and limitations

A strength of this study was the utilization of the HBM to gain a deep understanding of community perspectives on child drowning and its seriousness within their communities. This, coupled with insights into risk factors, some of which are specific to the Indonesian context, is an important starting point for further research into contextually relevant health promotion approaches to address child drowning.

There are, however, some limitations that need to be acknowledged. This study was conducted on just one Indonesian island, although the sampling approach ensured a diverse geographical representation. Indonesia’s vast diversity necessitates further research across the country to gauge if perceptions of drowning and risk factors align consistently. In addition, participants self-selected into the study, which may bias the sample towards those with a particular interest in or experience in drowning. This could result in findings that do not fully represent the broader community’s views. Furthermore, parents and village community leaders were grouped in the same focus groups, hence it was not always possible to distinguish between their perspectives. This potential overlap in perspectives due to the composition of the focus group may have influenced the interpretation of findings, limiting the ability to compare perspectives distinctly between parents and village community leaders.

## CONCLUSION

This qualitative study identified limited community understanding of the preventability, vulnerability and risk factors of child drowning. Utilizing constructs from the HBM, this study revealed that most participants were unaware of the susceptibility of their children and others in the community to drowning, and of the potential severe outcomes of drowning such as injury, disability and death. This highlights the urgent need to enhance public awareness about the susceptibility of children to drowning and the severe consequences it can entail. While direct exposure to drowning incidents prompted some awareness of the issue, most participants were in the early stages of behavioural change regarding protective strategies. Further exploration of local contexts and social determinants of drowning in Indonesian communities is imperative for ensuring effective and sustainable water safety promotion and drowning prevention strategies.

## Data Availability

The direct and anonymized quotes supporting this article are available within the article itself. Complete transcripts cannot be shared publicly due to privacy considerations for the study participants, in accordance with ethical approval. All anonymized transcripts are stored at the James Cook University Research Data Management Repository, in line with ethical approval protocols.
